# A stromal cell niche sustains ILC2-mediated type-2 conditioning in adipose tissue

**DOI:** 10.1084/jem.20190689

**Published:** 2019-06-27

**Authors:** Batika M.J. Rana, Eric Jou, Jillian L. Barlow, Noe Rodriguez-Rodriguez, Jennifer A. Walker, Claire Knox, Helen E. Jolin, Clare S. Hardman, Meera Sivasubramaniam, Aydan Szeto, E. Suzanne Cohen, Ian C. Scott, Matthew A. Sleeman, Chiamaka I. Chidomere, Sara Cruz Migoni, Jorge Caamano, Helle F. Jorgensen, Stefania Carobbio, Antonio Vidal-Puig, Andrew N.J. McKenzie

**Affiliations:** 1 Medical Research Council Laboratory of Molecular Biology, Cambridge, UK; 2 Department of Respiratory, Inflammation and Autoimmunity, AstraZeneca, Cambridge, UK; 3 College of Medical and Dental Sciences, Institute of Immunology and Immunotherapy, University of Birmingham, Birmingham, UK; 4 Cardiovascular Medicine Division, Department of Medicine, University of Cambridge, Cambridge, UK; 5 Wellcome Trust Sanger Institute, Hinxton, UK; 6 Metabolic Research Laboratories, Addenbrooke's Treatment Centre, Institute of Metabolic Science, Addenbrooke's Hospital, University of Cambridge, Cambridge, UK

## Abstract

Group-2 innate lymphoid cells (ILC2), type-2 cytokines, and eosinophils have all been implicated in sustaining adipose tissue homeostasis. However, the interplay between the stroma and adipose-resident immune cells is less well understood. We identify that white adipose tissue–resident multipotent stromal cells (WAT-MSCs) can act as a reservoir for IL-33, especially after cell stress, but also provide additional signals for sustaining ILC2. Indeed, we demonstrate that WAT-MSCs also support ICAM-1–mediated proliferation and activation of LFA-1–expressing ILC2s. Consequently, ILC2-derived IL-4 and IL-13 feed back to induce eotaxin secretion from WAT-MSCs, supporting eosinophil recruitment. Thus, MSCs provide a niche for multifaceted dialogue with ILC2 to sustain a type-2 immune environment in WAT.

## Abstract (precis)

Rana et al. demonstrate that white adipose tissue-resident multipotent stromal cells (WAT-MSC) support IL-33– and LFA-1/ICAM-1–mediated proliferation and type-2 cytokine expression by ILC2. Inversely, ILC2-derived IL-4 and IL-13 promote WAT-MSC eotaxin production and eosinophil recruitment, further maintaining type-2 immune homeostasis.

## Graphical Abstract

**Figure:**
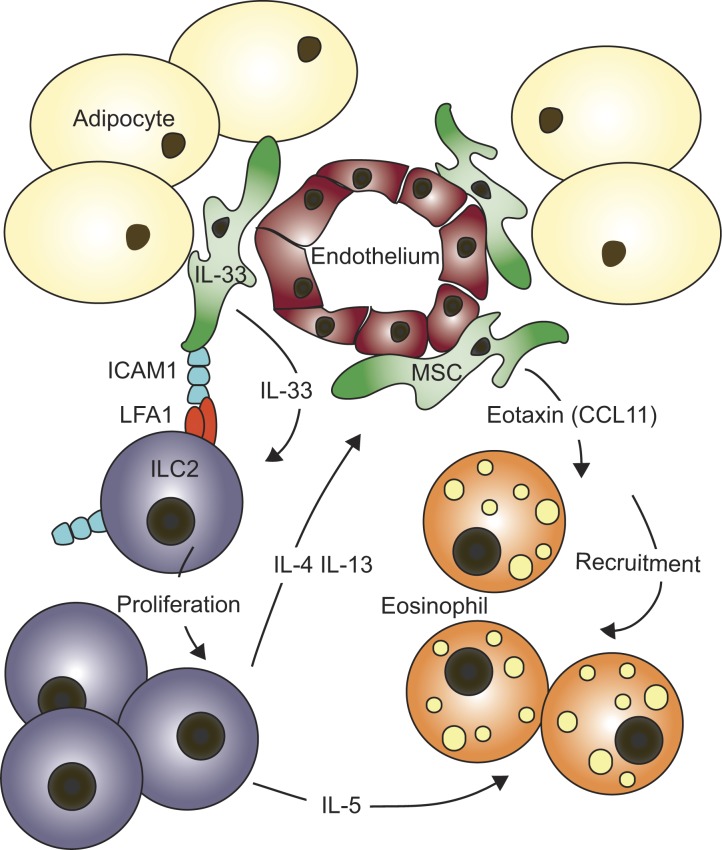

## Introduction

Group-2 innate lymphoid cells (ILC2s) respond rapidly at mucosal surfaces to combat infection but also contribute to the maintenance of tissue repair and homeostasis (Vivier et al., 2018). Signals within the tissue microenvironment help dictate the phenotype of resident ILC2s, equipping them with attributes commensurate with the physiological requirements of their location (Ricardo-Gonzalez et al., 2018). White adipose tissue (WAT)–resident ILC2s contribute to the maintenance of metabolic homeostasis (Molofsky et al., 2013; Brestoff et al., 2015; Lee et al., 2015), and receive signals from adipocytes and stromal vascular fraction (SVF)–derived cells (Dykstra et al., 2017; Dahlgren et al., 2019; Mahlakõiv et al., 2019). Notably, lean WAT is distinguished by a type-2 immune environment populated by alternatively activated M2 macrophages, eosinophils, ILC2, regulatory T cells, and cytokines including IL-4, IL-5, IL-13, and IL-33 (Cipolletta et al., 2012; Bapat et al., 2015; Molofsky et al., 2015; Schwartz et al., 2016; Lee et al., 2018). By contrast, low-grade type-1 inflammation, characterized by classically activated M1 macrophages, Th1 cells, and cytokines including IL-1β, IL-18, TNFα, and IL-8, is associated with increased obesity (Weisberg et al., 2003; Odegaard and Chawla, 2015; Schwartz et al., 2016; Kumari et al., 2018).

In mice, administration of IL-33 induced ILC2 activation and promoted beiging of adipocytes, a process associated with increased metabolic consumption (Brestoff et al., 2015; Lee et al., 2015). A number of mechanisms appear to underlie these changes. ILC2s were shown to produce the endogenous opioid peptide Met-enkephalin that may contribute to regulating obesity (Brestoff et al., 2015). Others reported that ILC2, along with eosinophils (Wu et al., 2011), produce IL-4 and/or IL-13, which directly promote beiging of adipocyte progenitors (Lee et al., 2015). IL-5–producing ILC2s were also required to sustain IL-4–secreting eosinophils in visceral adipose tissue (Molofsky et al., 2013), and mice lacking eosinophils gained more weight (Wu et al., 2011). These studies support key roles for IL-33, ILC2, and eosinophils in regulating a lean phenotype.

Here, we aim to clarify the stromally elicited signals sustaining a type-2 immune microenvironment in healthy adipose tissue homeostasis by interrogating the mechanisms by which multipotent stromal cells (MSCs) and ILC2s communicate within this niche.

## Results and discussion

**Figure 1.**
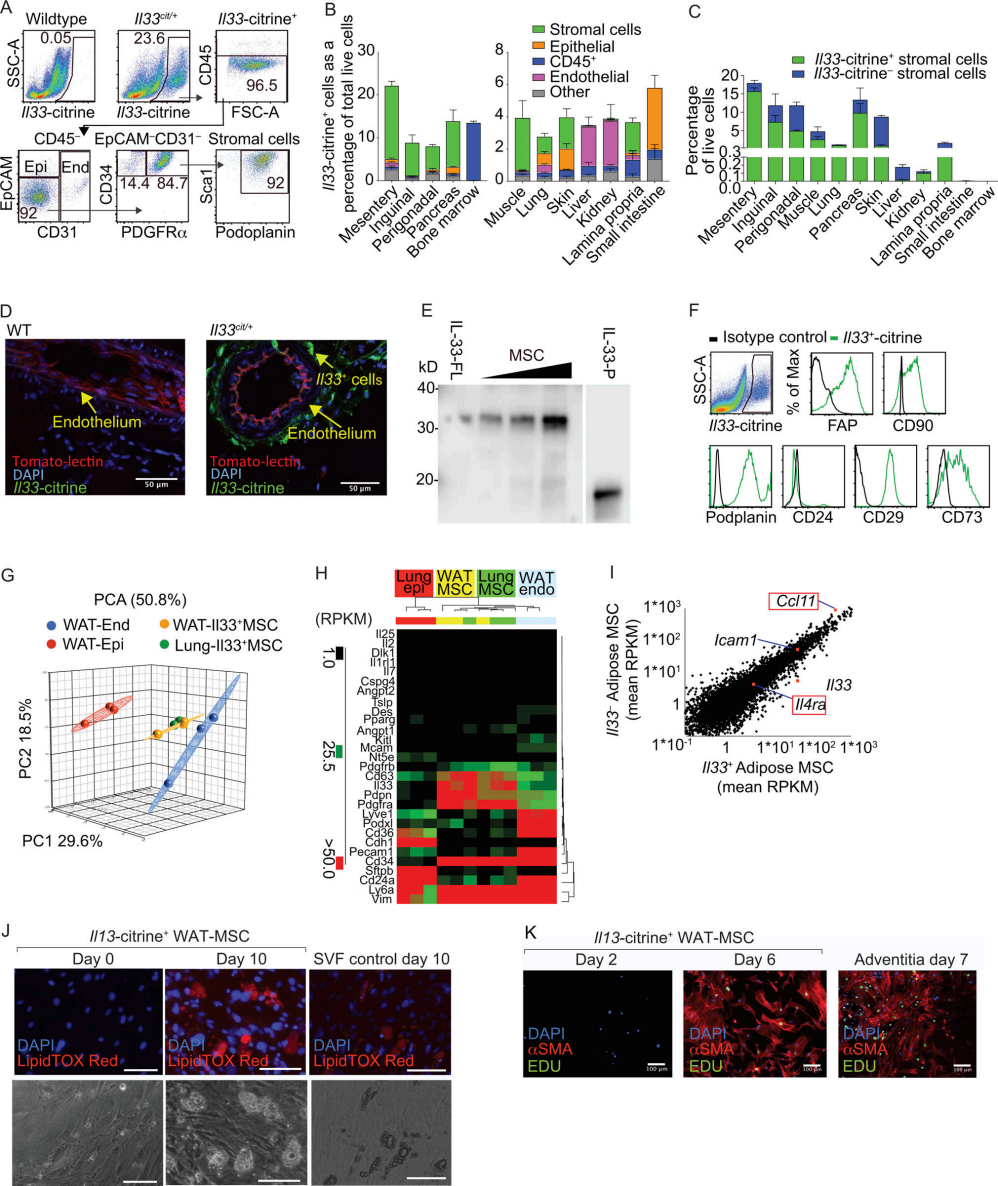
**Adipose resident IL-33^+^ cells are MSCs. (A)** Gating strategy. CD45^–^EpCAM^+^ epithelial cells (Epi), CD45^–^CD31^+^ endothelial cells (End), and CD45^–^EpCAM^–^CD31^–^PDGFRα^+^CD34^+^ stromal cells. SSC-A, side scatter area; FSC-A, forward scatter area. **(B)** Proportion of *Il33-*citrine–positive cells in tissues from *Il33^cit/+^* mice (*n* = 4, representative of two similar independent experiments). **(C)** Proportion of *Il33-*citrine*^+^*^or–^CD45^–^EpCAM^–^PDGFRα^+^ stromal cells among live cells in indicated tissues (*n* = 4). **(D)** Histology of WT or *Il33^cit/+^* mesentery: tomato lectin stain of capillary lumen. Scale bars, 50 µm. **(E)** Western blot analysis of IL-33 protein from purified WAT-MSCs. Full-length mouse IL-33 (IL-33-FL) in lysate of HEK cells expressing recombinant IL-33 and truncated mouse IL-33 (processed, IL-33-P). Representative of two similar independent experiments. **(F)** Phenotyping of *Il33-*citrine^+^ stromal cells. **(G)** Principal component analysis (PCA) of RNA-seq data from indicated cell populations (*n* = 3). **(H)** Gene expression data (reads per kilobase of transcript per million mapped reads; RPKM). Representative of at least two repeat experiments. **(I)** Comparison of *Il33*^+^ and *Il33*^–^ WAT-MSCs from adipose tissue. Genes of interest are highlighted (*n* = 3). **(J)** Adipose differentiation determined by lipid droplet analysis. Representative of two experiments. Scale bars, 100 µm. **(K)** Myocyte differentiation determined by α-smooth muscle actin (αSMA) staining. Representative of three experiments. Scale bars, 100 µm. Data are represented as mean ± SEM. Max, maximum.

Consistent with other data (Molofsky et al., 2013), WAT was enriched with IL-33–receptor ST2^hi^ ILC2s (Fig. S1, A–E), and ILC2-deficient (*Rora^fl/fl^Il7ra^cre^*) mice displayed markedly reduced WAT IL-13 and IL-5 expression and decreased eosinophil frequency (Fig. S1, F–L). Given the prominent role of IL-33 in supporting ILC2 proliferation, we used *Il33*-citrine reporter mice (Hardman et al., 2013) to identify stromal cells as the major source of *Il33*-citrine expression in WAT, constituting ∼87% of CD45^–^PDGFRα^+^ mesenteric WAT stromal cells, ∼62% in the inguinal adipose tissue, and ∼41% in the perigonadal fat pad (Fig. 1, A–C; and Fig. S1 M). These *Il33*-citrine^+^ cells were located around the endothelial layer in the mesentery (Fig. 1 D), a position akin to that reported for mesenchymal stem cells or pericytes (Corselli et al., 2010; Dahlgren et al., 2019); and surrounding fat-associated lymphoid clusters (Fig. S1 N). Western blot analysis of purified WAT stromal cells confirmed their expression of full-length IL-33 (Fig. 1 E).

Flow cytometry, gene expression profiles, and principal component analysis established that adipose-resident CD45^–^PDGFRα^+^*Il33*-citrine^+or–^ stromal cells (CD45^–^FAP^+^CD90^+^podoplanin^+^EpCAM^–^CD24^–^*Tie2*-GFP^–^CD29^+^CD73^+^CD34^+^Sca1[Ly6a]^+^CD63^+^vimentin[Vim]^+^) were distinct from other known IL-33–expressing populations: epithelial cells (CD45^–^CD31^–^EpCAM^+^E-cadherin[Cdh1]^+^ surfactant protein B[Sftbp]^+^); endothelial cells (CD45^–^CD31^+^EpCAM^–^CD36^+^podocalyxin[Podxl]^+^lymphatic vessel endothelial hyaluronan receptor 1[Lyve1]^+^); pericytes (Dlk1^+^Cspg4^+^CD146[Mcam]^+^Pparg^+^angiopoietin-1 and -2[Angpt1 and 2]^+^); and PDGFRα^–^CD31^+^*Tie2*-GFP^+^ endothelial progenitors (Fig. 1, F–H; and Fig. S2, A–C). Gene expression for known ILC2-activating factors including *Il7*, *Il2*, *Il25*, *Tslp*, and *Kitl* (SCF), as well as *Il1rl1* (IL-33 receptor, ST2), was negligible in PDGFRα^+^*Il33*-citrine^+or–^ WAT stromal cells (Fig. 1 H and data not shown). These data suggested that IL-33 was the predominant ILC2-inducing cytokine produced by WAT stromal cells, and unlike a previous report, we were unable to detect TSLP, perhaps due to the differences between WAT and lung stromal cells (Dahlgren et al., 2019). Although we identified *Il33*-citrine^+or–^ WAT stromal cells these were found to be highly similar with respect to phenotype and gene expression (Fig. 1 I).

WAT stromal cells have a gene expression profile characteristic of MSCs, which have the ability to differentiate into multiple cell lineages (Cawthorn et al., 2012; Farahani and Xaymardan, 2015). Indeed, PDGFRα^+^*Il33*-citrine^+or–^ stromal cells cultured in vitro with defined adipogenic factors readily differentiated into LipidTOX Red–staining lipid-storing mature adipocytes (Fig. 1 J and data not shown), similar to a positive control constituting the SVF from perigonadal adipose tissue (Fig. 1 J). Furthermore, PDGFRα^+^*Il33*-citrine^+^ WAT stromal cells also possessed myogenic potential when cultured in vitro with PDGF and TGFβ, giving rise to proliferating 5-ethynyl-2′-deoxyuridine (EdU)^+^ α-smooth muscle actin–positive cells in culture, resembling a positive control from aorta adventitia (Fig. 1 K). Consequently, we subsequently refer to these cells as WAT-MSCs. Recently, IL-33 expression has been reported in stromal cells in multiple tissues (Heredia et al., 2013; Kuswanto et al., 2016; Dalmas et al., 2017; Dahlgren et al., 2019; Mahlakõiv et al., 2019; Spallanzani et al., 2019), suggesting that similar cells may represent an important source of IL-33 for maintaining homeostasis and repair throughout the body.

**Figure 2.**
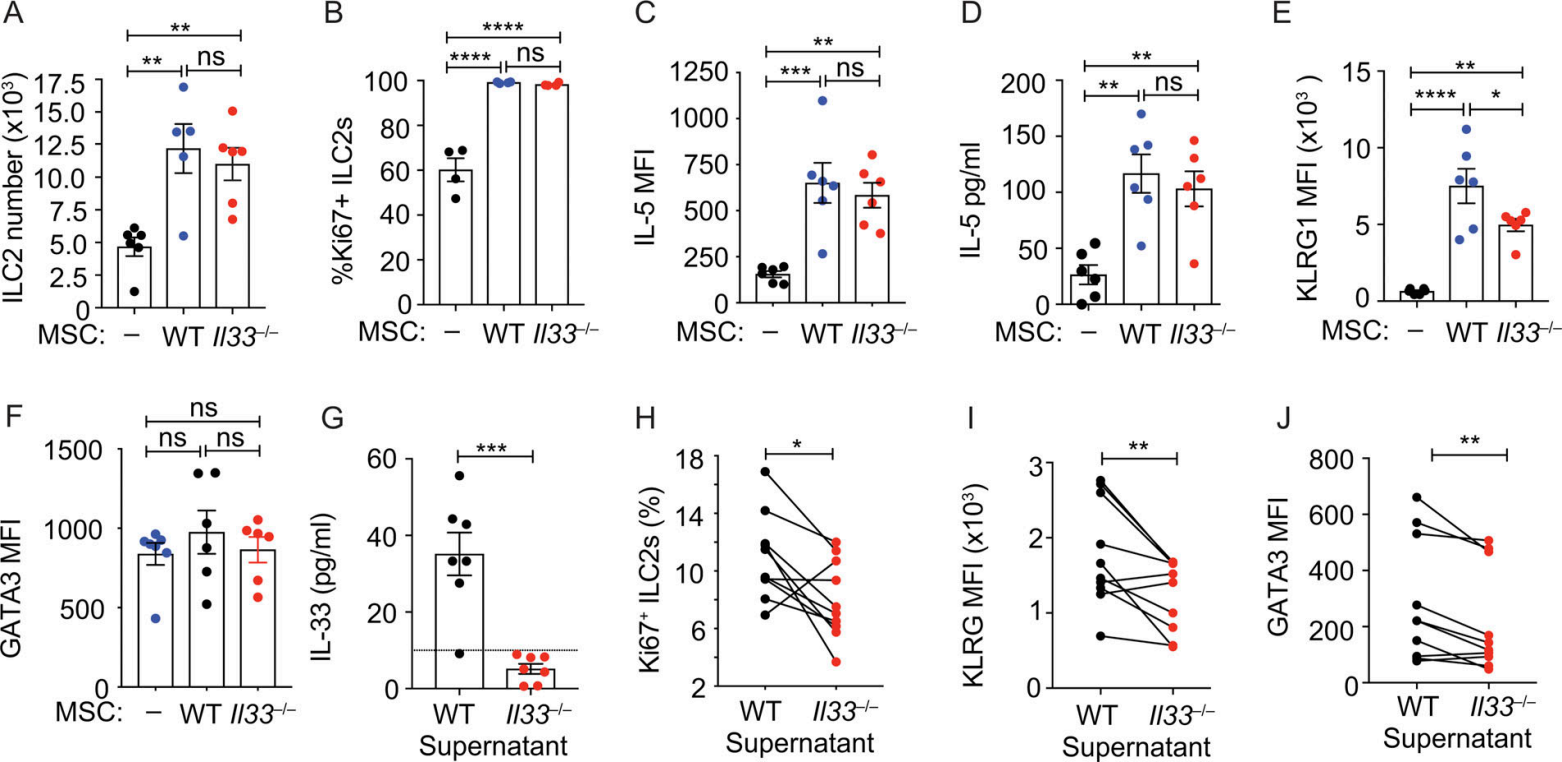
**ILC2s respond to MSC-derived IL-33. (A)** ILC2 number at 7 d of culture with WT or IL-33–deficient (*Il33*^−/−^) MSCs. Pooled data from three experiments (*n* = 5 or 6). **(B)** Frequency of Ki67^+^ILC2 in co-cultures at day 7. One of two similar experiments (*n* = 4). **(C)** Mean fluorescent intensity (MFI) of intracellular IL-5 expression by ILC2s in co-cultures at day 7, determined by flow cytometry. **(D)** IL-5 in co-culture supernatants at day 7 determined by ELISA. **(E and F)** MFI of KLRG1 (E) or of intracellular GATA3 expression by ILC2s from co-cultures at day 7 (F). Pooled data are from two experiments (*n* = 6 mice; C–F). **(G)** IL-33 in freeze-thawed SVF supernatants analyzed by ELISA. Pooled data from three experiments (*n* = 7). **(H)** Frequency of Ki67^+^ ILC2s cultured for 48 h with supernatants from I. Pooled data represent 10 separate ILC2 purifications from three independent experiments. **(I and J)** MFI of KLRG1 (I) or GATA3 (J) expression by ILC2s, cultured as in H. Pooled data represent 10 separate ILC2 purifications from three independent experiments. Data are mean ± SEM. ns, not significant; *, P < 0.05; **, P < 0.01; ***, P < 0.001; ****, P < 0.0001; statistical analysis, one-way ANOVA with Tukey’s post hoc test (A–F), Student’s *t* test (G), or paired Student’s *t* test (H–J).

Given the conserved phenotype and gene expression of *Il33*-citrine^+and–^ WAT-MSCs, we subsequently used total WAT-MSCs, independent of their *Il33*-citrine expression, to assess their potential to regulate ILC2 biology. We found that co-culture of WAT-MSCs with naive WAT-ILC2s (Fig. S3, A and B), induced ILC2 proliferation (Fig. 2, A and B) and IL-5 expression, as compared with cultures lacking WAT-MSCs (Fig. 2, C and D). Unexpectedly, these effects were predominantly IL-33–independent, as ILC2 proliferation was largely unchanged when IL-33–deficient WAT-MSCs were used in this assay (Fig. 2, A–D). We also excluded a potential role for contaminating MSCs by confirming the result using both IL-33–deficient ILC2 and MSCs (Fig. S3 C). Nevertheless, we did observe that MSC-derived IL-33 was required to elicit up-regulation of the ILC2 activation marker KLRG1 in two of three experiments, but not GATA3 (Fig. 2, E and F). It has been proposed that cell stress and damage are required to trigger IL-33 release in vivo (Moussion et al., 2008; Scott et al., 2018). Consequently, to replicate in situ cell stress/damage, the SVF from WT or IL-33–deficient mice was freeze-thawed to induce IL-33 release (Fig. 2 G), and supernatants were added to ILC2 cultures. Supernatants from IL-33–deficient SVF were less efficient at stimulating ILC2 proliferation (Fig. 2 H), KLRG1 up-regulation (Fig. 2 I), and GATA3 expression (Fig. 2 J) than WT samples. Taken together, these results suggest that WAT-MSCs express IL-33 in healthy WAT and are capable of releasing IL-33 release upon cellular stress or damage. Indeed, a recent report has demonstrated that absence of IL-33 from PDGFRα^+^ stromal cells reduced the type-2 response in the lung following helminth infection (Dahlgren et al., 2019).

**Figure 3.**
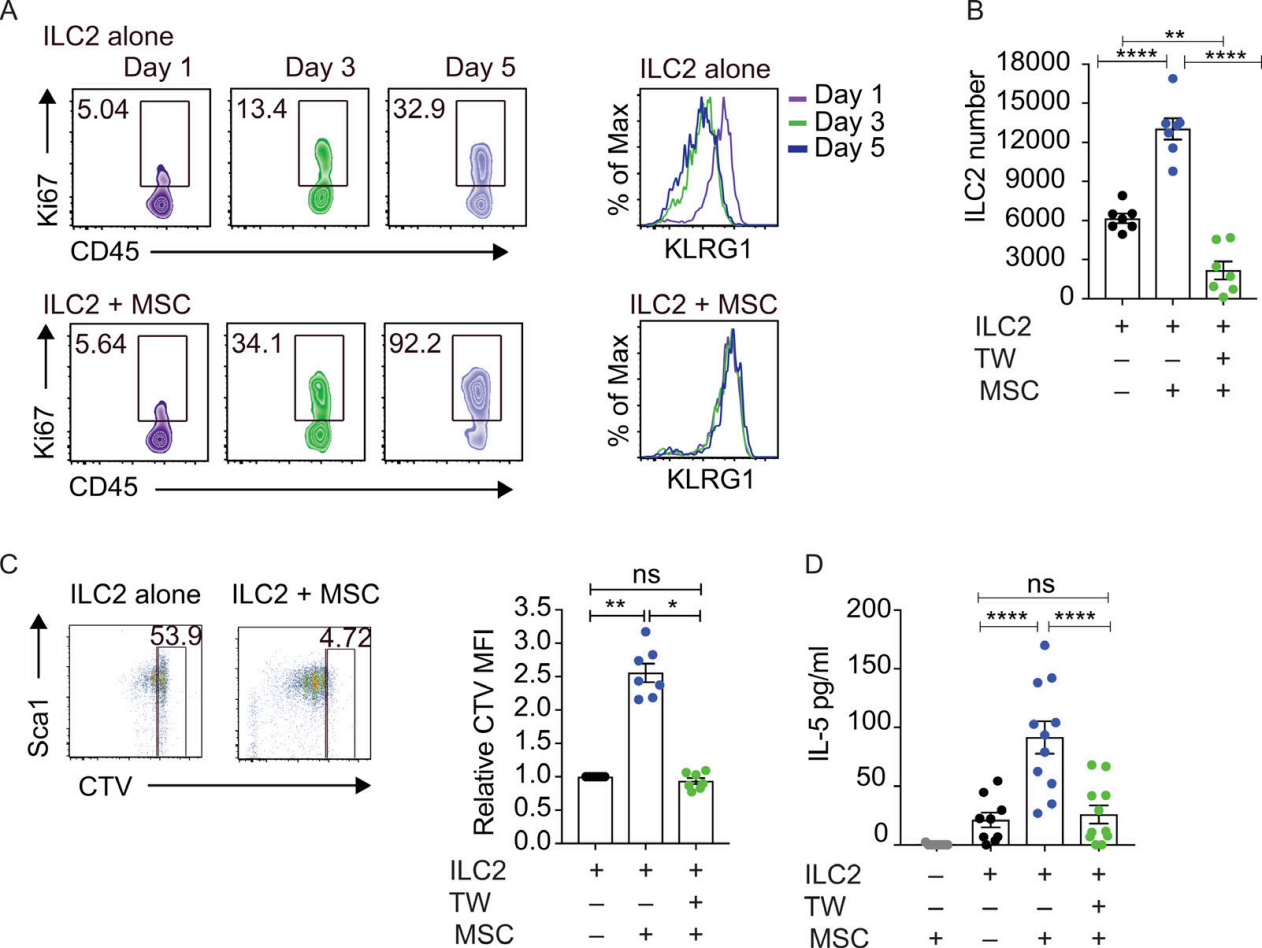
**WAT-MSCs induce ILC2 proliferation and type-2 cytokine production. (A)** Frequency of Ki67^+^ILC2s and KLRG1 expression by ILC2 from ILC2 alone, or ILC2 cultured with MSCs (ILC2 + MSCs). **(B)** ILC2 cell counts after 7 d of co-culture. TW, transwell. Pooled data from two experiments (*n* = 7). **(C)** Flow cytometric analysis of CTV dilution and quantification of relative CTV MFI (CTV MFI sample/CTV MFI ILC2)^−1^ after 7 d of co-culture. Pooled data from two experiments (*n* = 7). **(D)** IL-5 in supernatants after 7 d of co-culture, as determined by ELISA. Pooled data from four experiments (*n* = 11). Data are mean ± SEM. ns, not significant; *, P < 0.05; **, P < 0.01; ****, P < 0.0001; statistical analysis, one-way ANOVA with Freidman test (C) or Tukey’s post hoc test (B and D).

However, even in the absence of IL-33, we observed that MSCs provided additional signals to support a type-2 immune microenvironment, results supported by the fact that depletion of IL-33^+^ MSCs, even with supplementation of exogenous IL-33, causes a reduction in the proportion of ILC2 in the lung (Dahlgren et al., 2019). To identify the IL-33–independent pathway through which WAT-MSCs regulate ILC2 proliferation, we determined if the factor was secreted or cell-bound. Time-course analysis confirmed the enhanced proliferation of ILC2 co-cultured in the presence of MSCs over 5 d, accompanied by the up-regulation of the activation marker KLRG1 (Fig. 3 A).

Co-culture of purified WAT-MSCs with ILC2 resulted in increased total cell counts (Fig. 3 B), which were ablated when the ILC2 and MSCs were separated using transwells (Fig. 3 B), though in part, this was due to the reduced survival of ILC2 on the transwell filter (Fig. S3 D). However, we also observed enhanced ILC2 proliferation, as determined by cell trace violet (CTV) staining (Fig. 3 C), which was reversed in the transwell culture, and was independent of the substrate on which they were cultured (Fig. 3 C). Similarly, MSC-induced, ILC2-derived IL-5 production was also reduced when the cells were separated in transwell assays (Fig. 3 D).

**Figure 4.**
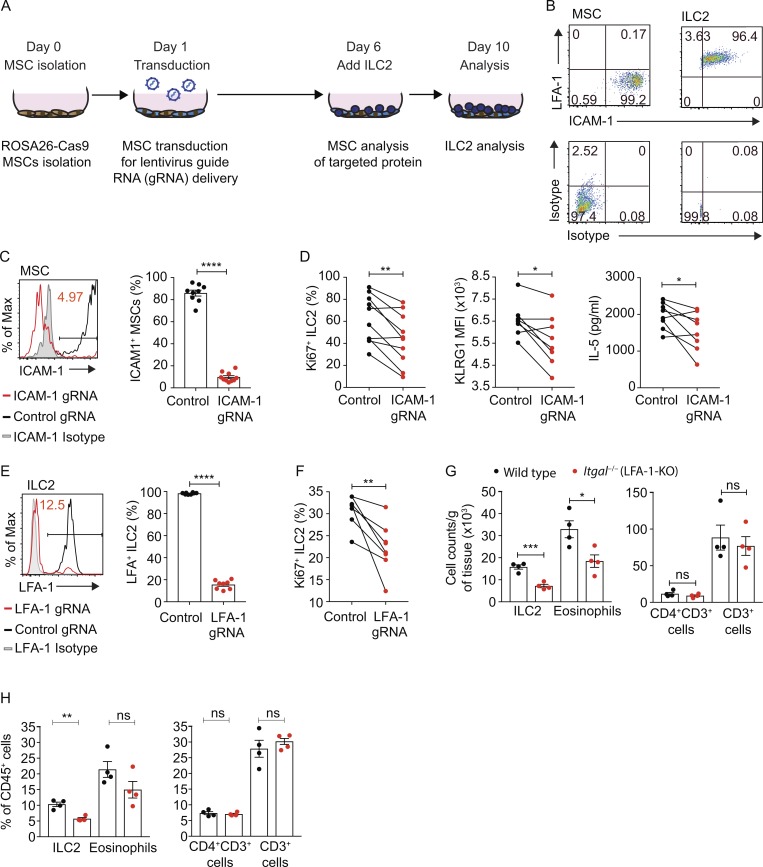
**Ligation of LFA-1 on ILC2 by ICAM-1 on WAT-MSCs induces ILC2 proliferation and IL-5 production. (A)** Schematic of CRISPR targeting knockout co-culture assay. **(B)** Representative flow cytometric analysis of ICAM-1 and LFA-1 expression by WAT-MSCs and ILC2. Data representative of two independent experiments (*n* = 6). **(C)** Representative example of MSC ICAM-1 CRISPR-Cas9–mediated knockdown. Non-targeting gRNA (Control gRNA); ICAM-1 targeting gRNA (ICAM-1 gRNA). Pooled data from four independent experiments (*n* = 9 or 10). **(D)** Frequency of Ki67^+^ILC2, KLRG1 MFI on ILC2s, and IL-5 concentrations (ELISA) from MSC ICAM-1–targeted cultures at day 10. Pooled data from 8–10 individual MSC purifications from at least two independent experiments (*n* = 8–10). **(E)** Representative example of LFA-1 CRISPR-Cas9–mediated knockdown. Non-targeting gRNA (Control gRNA); LFA-1 targeting gRNA (LFA-1 gRNA). Pooled data from two experiments (*n* = 8 mice). **(F)** Frequency of Ki67^+^ILC2 from ILC2 LFA-1–targeted cultures at day 10. Pooled data from eight individual MSC purifications from two independent experiments (*n* = 8). **(G and H)** Cell numbers (G) and frequency (H) of ILC2, eosinophil, CD4^+^CD3^+^ T cell, and CD3^+^ T cell in combined perigonadal and inguinal adipose tissue. Control (WT) and *Itgal*^−/−^ (LFA-1-KO) mice. Data representative of two independent experiments (*n* = 4 mice). Data are mean ± SEM. ns, not significant; *, P < 0.05; **, P < 0.01; ****, P < 0.0001; statistical analysis, Student’s *t* tests (C, E, G, and H) or paired Student’s *t* tests (D and F).

To isolate the cell surface–associated proliferative/maintenance signal, we interrogated WAT-MSC and WAT-ILC2 gene expression data for potential ligand/counter-ligand pairs. CRISPR-Cas9–mediated candidate gene deletion was performed in MSCs derived from Cas9-transgenic mice using lentiviral vectors carrying target gene–specific guide RNAs (gRNAs; Fig. 4 A). With this approach, we determined that intercellular adhesion molecule-1 (ICAM-1) was expressed on WAT-MSCs while its counter-ligand LFA antigen-1 (LFA-1) was present on WAT-ILC2s (Fig. 4 B). ICAM-1 and LFA-1 facilitate lymphocyte accumulation at sites of immune activation where ICAM-1 is up-regulated on stromal cells (Verma and Kelleher, 2017; Walling and Kim, 2018) but can also initiate LFA-1 signaling leading to T cell activation and proliferation (Verma and Kelleher, 2017; Walling and Kim, 2018) and promote Th1 cell differentiation of human T cells (Smits et al., 2002). In addition, LFA-1 ligation has been described to mediate TCR-independent signals, such as respiratory burst induction in neutrophils, suggesting that LFA-1 can serve as an activating receptor in cells by itself (Berton et al., 1992). ICAM-1 was efficiently deleted from MSCs (Fig. 4 C). Notably, co-culture of ICAM-1 gRNA-targeted MSCs with WT ILC2 resulted in impaired ILC2 proliferation, reduced KLRG1 expression, and reduced IL-5 production as compared with controls (Fig. 4 D). To validate this ICAM-1–mediated interaction, we assessed the involvement of the reciprocal LFA-1 ligand by CRISPR-Cas9–targeting in ILC2s. Knockdown of LFA-1 expression on ILC2 was highly efficient, compared with control gRNA (Fig. 4 E). Furthermore, ILC2 lacking LFA-1 expression failed to proliferate as efficiently as control gRNA-targeted ILC2 when cultured with WT MSCs (Fig. 4 F). Thus, an ICAM-1/LFA-1 receptor–mediated interaction between MSCs and ILC2 enhances ILC2 proliferation.

We next examined the frequency of ILC2 in WAT from naive LFA-1–deficient (*Itgal*^−/−^) mice and found that ILC2 numbers and frequencies were significantly lower in the absence of LFA-1 (Fig. 4, G and H; and Fig. S3 E). Furthermore, this deficit was mirrored by a decrease in WAT eosinophils (Fig. 4, G and H; and Fig. S3 E). By contrast, the proportion and number of CD4^+^ T cells and CD3^+^ cells in the WAT were not altered by the deletion of LFA-1 (Fig. 4, G and H; and Fig. S3 E). Thus, the absence of LFA-1 in vivo leads to a diminution of ILC2s in adipose tissue, and this correlated with fewer eosinophils. Our data suggest that inefficient ILC2 proliferation may contribute to the observed ILC2 deficit, but selective cellular adhesion may also be important for localization and retention of ILC2 in WAT. *Icam1*^−/−^ mice have been reported to spontaneously gain weight when maintained on normal diet (Dong et al., 1997), and it is possible, given our data, that the interaction of ICAM-1 with LFA-1 on ILC2s contributes to this phenotype. Interestingly, a recent report has indicated that signaling via ICAM-1 expressed on common lymphoid progenitors promotes their differentiation to ILC2s, and may contribute to the proliferation of ICAM-1^+^ ILC2 in response to IL-33 (Lei et al., 2018). Furthermore, stromal cells in the mesentery supported ILC2 differentiation from ILC precursors (Koga et al., 2018). However, although we also detected ICAM-1 on ILC2, the effect on proliferation that we observed was dependent on ICAM-1 present on the MSCs and LFA-1 expression by ILC2s. In combination, these findings indicate the importance of future studies to understand how the LFA-1 and ICAM-1 signaling pathways, such as calcium flux and PI3K activation, modulate ILC2 function (Berton et al., 1992; Verma and Kelleher, 2017).

**Figure 5.**
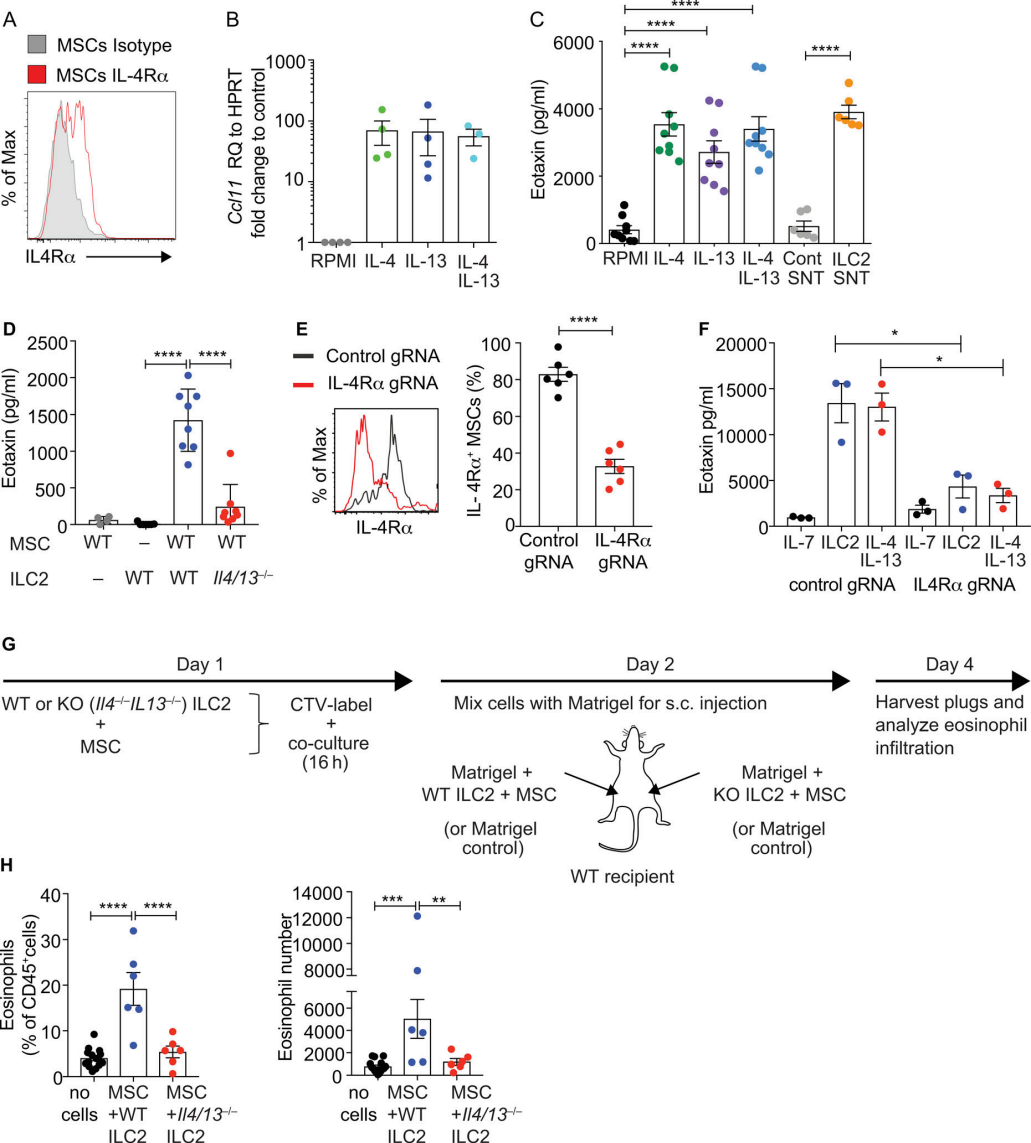
**ILC2 and MSCs coordinate eosinophil recruitment in vivo. (A)** Representative flow cytometric analysis of IL-4Rα expression by WAT-MSCs. **(B)** Quantitative PCR analysis of eotaxin (*Ccl11*) expression by purified WAT-MSCs cultured with the indicated cytokines for 48 h. Pooled data from three experiments (*n* = 4). RQ, relative quantification; HRPT, hypoxanthine phosphoribosyltransferase. **(C)** ELISA detection of eotaxin in supernatants (at 7 d) from WAT-MSCs and ILC2 co-cultures treated as indicated. SNT, supernatant. Pooled data from three to five independent experiments (*n* = 6–9). **(D)** Detection of eotaxin in supernatants from MSCs and ILC2s (WT, WT BALB/c, or *Il4*^−/−^/*Il13*^−/−^ BALB/c) cultured as indicated for 7 d, as determined by ELISA. Pooled data from two experiments (*n* = 4–8). **(E)** Representative example of IL-4Rα CRISPR-Cas9–mediated knockdown. Non-targeting gRNA (Control gRNA); IL-4Rα targeting gRNA (IL-4Rα gRNA; *n* = 6). Representative of two similar experiments. **(F)** ELISA detection of eotaxin in supernatants from MSCs transduced with lentiviral vectors containing non-targeting gRNA (Control gRNA) or IL-4Rα targeting gRNA (IL-4Rα gRNA), and cultured with ILC2 or recombinant IL-13 and IL-4 for 4 d (*n* = 3 mice). Representative of two similar experiments. **(G)** Schematic of eosinophil recruitment model. **(H)** Flow cytometry analysis of CD45^+^GR1^–^CD11b^+^SiglecF^+^ eosinophil recruitment to Matrigel plugs harvested from WT recipients, shown as eosinophil percentage of CD45^+^ cells and absolute number of eosinophils per Matrigel plug. Pooled data from three experiments (*n* = 6 experimental, and *n* = 15 control). Data represented as mean ± SEM. *, P < 0.05; **, P < 0.01; ***, P < 0.001; ****, P < 0.0001; statistical analysis, repeated-measures ANOVA with Tukey’s post hoc test (C and F), one-way ANOVA (B, D, and H), or Student’s *t* test (E).

Intriguingly, our RNA sequencing (RNA-seq) data, concordant with a recently published report (Dahlgren et al., 2019), identified that WAT-MSCs highly expressed the eosinophil chemo-attractant CCL11 (eotaxin; Fig. 1 I and Fig. S3 F), but not CCL24 (eotaxin-2) or CCL26 (eotaxin-3; data not shown), responsible for eosinophil migration to peripheral tissues (Wen and Rothenberg, 2016). This raised the possibility that MSCs might contribute to eosinophil recruitment to adipose tissue and that this may be regulated by ILC2s. For example, eotaxin is up-regulated through IL-4 receptor (IL-4Rα) signaling (Moore et al., 2002; Zhou et al., 2012; Lee et al., 2015; Gieseck et al., 2016), which is a component of the IL-4 and IL-13 receptor complexes, and is expressed by WAT-MSCs (Fig. 1 I and Fig. 5 A). Culture of WAT-MSCs in vitro with IL-4 or IL-13 induced eotaxin expression (Fig. 5, B and C). Furthermore, ILC2 cell-culture supernatant also drove eotaxin expression from WAT-MSCs, as compared with control supernatant (Fig. 5 C). This effect was dependent on WAT-ILC2–derived IL-4/IL-13 as ILC2s from *Il4*^−/−^*Il13*^−/−^ BALB/c mice failed to induce eotaxin secretion, as compared with BALB/c WT controls (Fig. 5 D). CRISPR-mediated IL-4Rα–gRNA targeted deletion of IL-4Rα on MSCs confirmed the requirement for IL-4Rα–mediated signaling (Fig. 5, E and F). We also investigated if IL-4 and IL-13 could regulate *Il33*-citrine expression by WAT-MSCs. Indeed, IL-4 and IL-13 treatment increased IL-33 expression (data not shown). Thus, ILC2-secreted IL-4 and IL-13 can prime the expression of the eosinophil-attracting chemokine, eotaxin, from WAT-MSCs and may provide a potential positive feedback loop for IL-33 expression from WAT-MSCs.

To evaluate whether the interaction of WAT-MSCs with IL-4/IL-13–producing ILC2 mediates eosinophil recruitment in vivo, WAT-MSCs, WT ILC2, or IL-4/IL-13–deficient ILC2s were purified, labeled with CTV, and co-cultured overnight, combined with Matrigel, and transferred subcutaneously into naive WT recipients (Fig. 5 G). After 48 h, the Matrigel plugs were isolated, and flow cytometry analysis revealed that eosinophils were increased when MSCs and WT ILC2 were cotransferred as compared with Matrigel alone (Fig. 5 H and Fig. S3 G). Notably, eosinophil recruitment was not observed when IL-4/IL-13–deficient ILC2s were combined with WAT-MSCs (Fig. 5 H), correlating with the absence of eotaxin (Fig. S3 H). IL-5 is known to promote eosinophil retention and survival in peripheral tissues, as well as acting as a growth and differentiation factor for eosinophil development in the bone marrow (Wen and Rothenberg, 2016), and maintaining eosinophils in adipose tissue (Molofsky et al., 2013). However, IL-5 is unlikely to influence these Matrigel assays as both WT and IL-4/IL-13–null ILC2s produce similar levels of IL-5 (data not shown). Together, these data highlight the synergy between ILC2s and WAT-MSCs and indicate the potential for ILC2-derived IL-4/IL-13–dependent induction of eotaxin production from MSCs to enhance eosinophil recruitment. IL-4/IL-13–deficient mice have been reported to have normal numbers of WAT eosinophils (Molofsky et al., 2013), though these mice do show eosinophil deficits in other tissues, especially upon immune challenge (Fallon et al., 2002). Furthermore, others have indicated that such a network may play a role in recruiting eosinophils to the liver during fibrosis (Gieseck et al., 2016). Further studies will be required to determine the potential role of this pathway in vivo during homeostasis and during dysregulation, and to understand the interplay between ICAM-1–LFA-1 signals and ILC2-derived IL-4/IL-13.

In summary, we show that adipose MSCs can help sustain the type-2 microenvironment by supporting the proliferation, activation, and type-2 cytokine production of ILC2, and by responding to ILC2-derived IL-4 and IL-13, up-regulate eotaxin to promote eosinophil recruitment. Thus, MSCs can provide a niche for the maintenance of type-2 immune homeostasis in adipose tissue.

## Materials and methods

### Mice

*Il4^−/−^Il13^−/−^* (McKenzie et al., 1999) mice were backcrossed on a BALB/c background for six generations, and control BALB/c mice were bred in-house. *Il7ra*^Cre^ (Schlenner et al., 2010), *Rora*^fl/fl^ (Oliphant et al., 2014), *Il33^cit/cit^* (Hardman et al., 2013), *Tie2*-GFP mice (Tg[TIE2GFP]287Sato/J; Jackson Laboratory), Rosa26-Cas9 knockin (Gt[ROSA]26Sor^tm1.1(CAG-cas9*,-EGFP)Fezh^; Jackson Laboratory), and *Itgal^−/−^* (*Itgal*^tm1Bll^; Jackson Laboratory) were on a C57Bl/6 background. C57Bl/6 Jax controls were bred in-house. All mice were maintained in the Medical Research Council ARES animal facility under specific pathogen–free conditions. All animal experiments undertaken in this study were done with the approval of the UK Home Office.

### Isolation of naive WAT-MSCs and naive WAT-ILC2 from C57BL/6 mice

WAT was mechanically dissociated in RPMI-1640, and digested with collagenase I (Life Technologies) and DNase I (Roche) at 37°C while shaking. Lin^–^CD45^–^EpCAM^–^CD31^–^CD24^–^PDGFRα^+^CD34^+^Sca1^+^podoplanin^+^ WAT-MSCs and lineage^–^ST2^+^EpCAM^–^ WAT-ILC2s were sorted by flow cytometry using Sony iCyt Synergy (Sony) to >95% purity. ILC2s were also isolated from mesenteric lymph node (MLN) as lineage^–^ST2^+^ cells. With the exception of the Matrigel study, all ILC2 and MSC assays were performed with cells isolated from naive C57BL/6 mice.

### Flow cytometry

Single cells were incubated with anti-mouse CD16/32 (BioXCell) to block Fc receptors and stained as indicated: PGDFRα PE (APA5; BioLegend), CD34 Alexa Fluor 660 (RAM34; eBioscience), podoplanin PECy7 (8.1.1; BioLegend), EpCAM (G8.8; BioLegend), Sca1 APC-Cy7 (D7; BioLegend), CD24 (M1/69; BioLegend), CD31 (390; BioLegend), CD29 Alexa Fluor 700 (HMBeta1-1; BioLegend), ST2 PerCP efluor710 (RM-ST2; eBioscience), CD4 BV785 (RM4-5; BioLegend), CD45 (bv510 30-F11; BioLegend), CD4 BV785 (RM4-5; BioLegend), ef780 live dead (eBioscience), SiglecF af647 (E50-2440; BD Biosciences), CD11b (M1/70; BioLegend), CD11c PE (N418; BioLegend), Ly6G PerCP efluor710 (1A8-Ly6g; eBioscience), IL-5 APC (TRFK5; BioLegend), IL-13 PE (eBio13A; R&D Systems), GATA3 BV711 (L50-823; BD Horizon), RORγt PE (AFKJS-9; eBioscience), FOXP3-PECy7 (FJK-16S; eBioscience), Lineage B220 (RA3-6B2), CD3 (17A2), CD4 (GK1.5), CD8s (53–6.7), CD11b (M1/70), CD11c (N418), CD19 (eBio1D3), FCεR1 (MAR-1), DX5 (DX5), F480 (BM8), GR1 (RB6-8C5), NK1.1 (PK136), TCRβ (H57-597), and Ter119 (TER-119; efluor450; eBioscience).

For intracellular and nuclear staining, cells were processed using the Foxp3/Transcription Factor Kit (eBioscience). For intracellular IL-5 and IL-13 detection, cells were cultured with PMA (50 ng/ml), ionomycin (500 ng/ml), and 1× protein transport inhibitor (eBioscience) in culture media, RPMI-1640, 10% FCS plus penicillin/streptomycin and 2-mercaptoethanol, for 4 h. Flow cytometry analysis was performed on a BD Fortessa instrument. Cell numbers were quantified based on precision count beads (BioLegend), and flow cytometry data were analyzed using FlowJo (version 9.9). Dead cells were excluded from analysis.

### RNA-seq

Cells were flow sorted into in RPMI-1640, 10% FCS plus penicillin/streptomycin, 2-mercaptoethanol and transferred to Trizol (Life Technologies). RNA was extracted using RNeasy kits and reagents (Qiagen). DNA was digested using Turbo DNase (Ambion). RNA was concentrated using an RNeasy Micro Kit (Qiagen) and assessed using a Bioanalyser (Agilent). RNA was processed for RNA-sequencing using an Ovation RNA-seq System V2 (Nugen), fragmented using the Covaris M220, and bar-coded using Ovation Ultralow Library Systems (Nugen). Samples were sequenced using an Illumina Hiseq4000 (Cancer Research UK Cambridge Institute), and sequence data were aligned using Tophat2 with Partek Flow. RNA-seq analysis was performed using Partek Genomics Suite software, version 6.16.

### In vitro cell culture and differentiation assays

For co-culture assays, 10,000 WAT-ILC2s were cultured with 100,000 WAT-MSCs (or at a ratio of 1:10 if fewer cells were available), in RPMI-1640, 10% FCS plus penicillin/streptomycin, 2-mercaptoethanol, and where applicable, polycarbonate transwells were used (0.4 µm; Scientific Laboratory Supplies). To maintain ILC2 survival, rmIL-7 (10 ng/ml; BioLegend) was included in all assays with ILC2s as well as controls, including when co-cultured with MSCs. The following cytokines were added to cultures as indicated: rmIL-13 (20 ng/ml; BioLegend), rmIL-4 (20 ng/ml; BioLegend), and rmIL-33 (10 ng/ml; BioLegend). IL-5 and eotaxin were detected by ELISAs (eBioscience and Peprotech, respectively), or eotaxin was analyzed using the MAGPIX assay (Luminex).

For adipocyte differentiation assays, total SVF from perigonadal fat was dispersed by digestion with collagenase type II before WAT–IL-33–citrine^+^MSCs were isolated by flow sorting. Cells were grown to confluence in growth media, high-glucose DMEM (glucose 25 mM; Sigma-Aldrich) and 1% penicillin/streptomycin (Sigma-Aldrich) with 10% neonatal calf serum. For adipose cell differentiation, cells were cultured for a further 10 d in growth media with 10% FBS supplemented with 3-isobutyl-1-methylxanthine (0.5 mM), dexamethasone (Sigma-Aldrich), and insulin (Sigma-Aldrich). LipidTOX Red staining (Thermo Fisher Scientific) was used to identify lipid droplets, and cultures were imaged using an EVOS Cell image system (Thermo Fisher Scientific).

For smooth muscle differentiation assays, cells were plated at 2,600 cells/cm^2^ and cultured with platelet-derived growth factor (PDGF)–BB (10 ng/ml; Peprotech) and TGFβ1 (2 ng/ml; Peprotech) in DMEM supplemented with 10% FCS, glutamine, penicillin, and streptomycin for 7 d. Growth factors were replenished every 2 d. Cells from aortic adventitia were treated in parallel as a positive control. To measure proliferation, EdU was added to the media 4 h before fixation in 4% formaldehyde. EdU detection was performed using the Click-it Plus 647 kit (c10640; Life Technologies) following the manufacturer’s instructions and differentiation assessed by staining with Alexa Fluor 594–conjugated anti–α-smooth muscle actin antibody (ab202368; Abcam) or control IgG (ab202368; Abcam).

### Ligand screen using CRISPR-Cas9 targeting

Lentiviral vectors were generated by transfecting HEK293(t) cells with TransIT-LT1 transfection reagent (Cambridge Bioscience) along with the plasmids psPAX2, pMD2.G, and lentiGuide-Puro (Addgene) that had been modified to express BFP and individual targeting gRNA (or random controls), designed according to the Broad GPP genome-wide Brie Library (Addgene). Lentivirus-containing supernatants were harvested and lentiviral particles concentrated using Lenti-X (Clontech). MSCs derived from the Cas9-expressing Rosa26-Cas9 knockin mice were plated on a flat-bottom 96-well plate (10^4^/well), and after 24 h transduced with respective lentiviral constructs. At day 6 of culture, purified ILC2s (5 × 10^3^) were added to the wells and cultured for a further 4 d (with IL-7 as described in In vitro cell culture and differentiation assays). At day 10 of culture, MSCs and ILC2 were analyzed by flow cytometry, and cell supernatants harvested for IL-5 ELISA.

Purified ILC2 from Cas9-expressing Rosa26-Cas9 knockin mice were plated on a round-bottom 96-well plate at 5 × 10^3^ ILC2 per well, and cultured for 2 d in complete RPMI (RPMI-1640, 10% FCS plus penicillin/streptomycin, 2-mercaptoethanol), and rmIL-7 and rmIL-33 (both interleukins at 10 ng/ml; BioLegend) before being transduced with respective lentiviral constructs. 4 d after transduction, Lin^–^CD45^+^BFP-positive ILC2s were purified using flow cytometry and plated on round-bottom 96-well plates, 5 × 10^3^ ILC2 per well. Target gene knockout was confirmed at this point with an aliquot of retrovirally transduced BFP-positive ILC2. Transduced ILC2s were then cultured for 3 d in complete RPMI with rmIL-7, but without rmIL-33 before co-culturing with MSCs for a further 4 d. MSCs (separately purified biological replicates) were flow-cytometrically sorted and plated in a flat-bottom 96-well plate 4 d before co-culturing with either target gene knockout or control transduced ILC2s. ILC2s were then harvested and analyzed by flow cytometry.

### Western analysis of IL-33

Tissues were homogenized in PBS with protease inhibitor 1× cOmplete, EDTA-free (Sigma-Aldrich), and analyzed by SDS-PAGE on NuPAGE Novex 12% Bis-Tris mini gels (Invitrogen) with 3-(N-morpholino)propanesulfonic acid (MOPS) running buffer (Invitrogen) according to the manufacturer’s instructions. All samples were reduced by heating to 95°C for 3 min in SDS-PAGE buffer containing 2% 2-mercaptoethanol. Proteins were transferred to nitrocellulose membranes (Invitrogen) and detected by Western blotting for mouse IL-33 using goat anti–IL-33 polyclonal antibody (AF3626; R&D Systems) at 1:1,000 dilution, as described previously (Scott et al., 2018). Immunoreactive proteins were identified with HRP-conjugated anti-goat (HAF109; R&D Systems) and Supersignal West Femto substrate (34095; Pierce).

### Cell imaging

Mice were perfused using 40 ml PBS vehicle control or 40 ml PBS containing 200 mg *Lycopersicon esculentum* (Tomato) lectin (Vector), followed by 40 ml PBS. Tissues were fixed in phosphate-buffered 1% methanol-free formaldehyde (Thermo Fisher Scientific), transferred to 30% sucrose, embedded in 15% sucrose + 7.5% porcine skin gelatin (Sigma-Aldrich) in PBS, and flash-frozen in isopentane at −80°C. 10-µM sections were mounted on Superfrost Plus slides (Thermo Fisher Scientific) and blocked using 1% donkey serum (Jackson Immunoresearch) in 0.05% Triton X-100 in PBS, and citrine was detected using polyclonal rabbit anti-GFP antibody (5 µg/ml; Life Technologies) followed by Alexa Fluor 488–labeled polyclonal donkey anti-rabbit antibody (4 µg/ml). Nuclei were stained using DAPI (300 nM) and coverslips mounted using Prolong Gold (Invitrogen). Sections were imaged using Zeiss 710. Anti–smooth muscle actin (ab202368; Alexa Fluor 594; Abcam) or IgG2a (ab178001; Alexa Fluor 594; Abcam) and EdU Click-iT kit (Thermo Fisher Scientific) were used to analyze smooth muscle differentiation assays and imaged using a Nikon HCA system.

For fat-associated lymphoid cluster analysis, tissues were fixed in 2% paraformaldehyde (Sigma-Aldrich), washed in PBS, and permeabilized with 1× Triton X-100 (Sigma-Aldrich) while shaking (Luckham Rotatest R100). Samples were washed, blocked using 10% horse serum, and incubated with primary antibodies at 4°C overnight on a gyratory rocker (Stuart). Samples were washed and incubated with a secondary antibody cocktail for 1 h on ice and stained with DAPI. Antibodies used for staining were anti-CD45 FITC (30-F11; eBioscience), then rabbit anti-FITC-488 (A-11090; Thermo Fisher Scientific); goat anti-IL-33 (AF3626; R&D Systems), then donkey anti-goat-555 (A-21432; Thermo Fisher Scientific); and rat anti-CD3-ef660 (17A2; eBioscience). Tissues were washed and mounted on slides with VectorShield mounting medium (Vector Labs). Confocal images of slides were obtained using the Zeiss LSM 780 confocal microscope.

### Matrigel adoptive transfers

WAT-MSCs were purified from naive BALB/c mice. ILC2s were purified from WT BALB/c or *Il4*^−/−^*Il13*^−/−^ BALB/c mice injected intraperitoneally with IL-25 and IL-2/anti–IL-2 complex (rmIL-2; 0.5 µg/mouse; BioLegend) and anti–IL-2 JES6-1A12 (0.25 µg/mouse; 2BScientific) preincubated at 37°C to form a complex and rmIL-25 (1 µg/mouse; Janssen) on 3 consecutive d. Isolated WAT-MSCs and ILC2s were co-cultured (MSCs + WT ILC2 or MSCs + IL-13/IL-4–deficient ILC2) at a ratio of 1:1 (100,000 of each) overnight in complete RPMI with IL-7 (10 ng/ml). The combined cells were then resuspended at 2 × 10^5^ per 100 µl of Matrigel (Phenol Red-Free Corning), and injected subcutaneously into either the left or right inguinal fat pad of the same mouse. Matrigel plugs were dissected 48 h later, mechanically dissociated in RPMI-1640, digested with collagenase I (Life Technologies) and DNase I (Roche) at 37°C while shaking, and passed through a filter to obtain a single-cell suspension for analysis by flow cytometry.

### Statistical analysis

Graphpad Prism software was used for all statistical analysis. RNA-seq analysis was performed using Partek Genomics Suite software, version 6.16.

### Data availability

The accession nos. for RNA sequencing datasets reported in this paper have been deposited with the Gene Expression Omnibus under accession no. GSE132738.

### Online supplemental material

Fig. S1 shows the ILC2 and MSC populations in the WATs. Fig. S2 shows gating strategies. Fig. S3 shows gating strategies and additional control experiments.

## Supplementary Material

Supplemental Materials (PDF)
