# Supplementary material for: A stromal cell niche sustains ILC2-mediated type-2 conditioning in adipose tissue
Source: J Exp Med. 2019 Jun 27;216(9):1999–2009. doi: 10.1084/jem.20190689 (PMC6719433; doi:10.1084/jem.20190689)
Supplement: Supplemental Materials (PDF) [file JEM_20190689_sm.pdf]

JEM

Supplemental material

Rana et al., https://doi.org/10.1084/jem.20190689

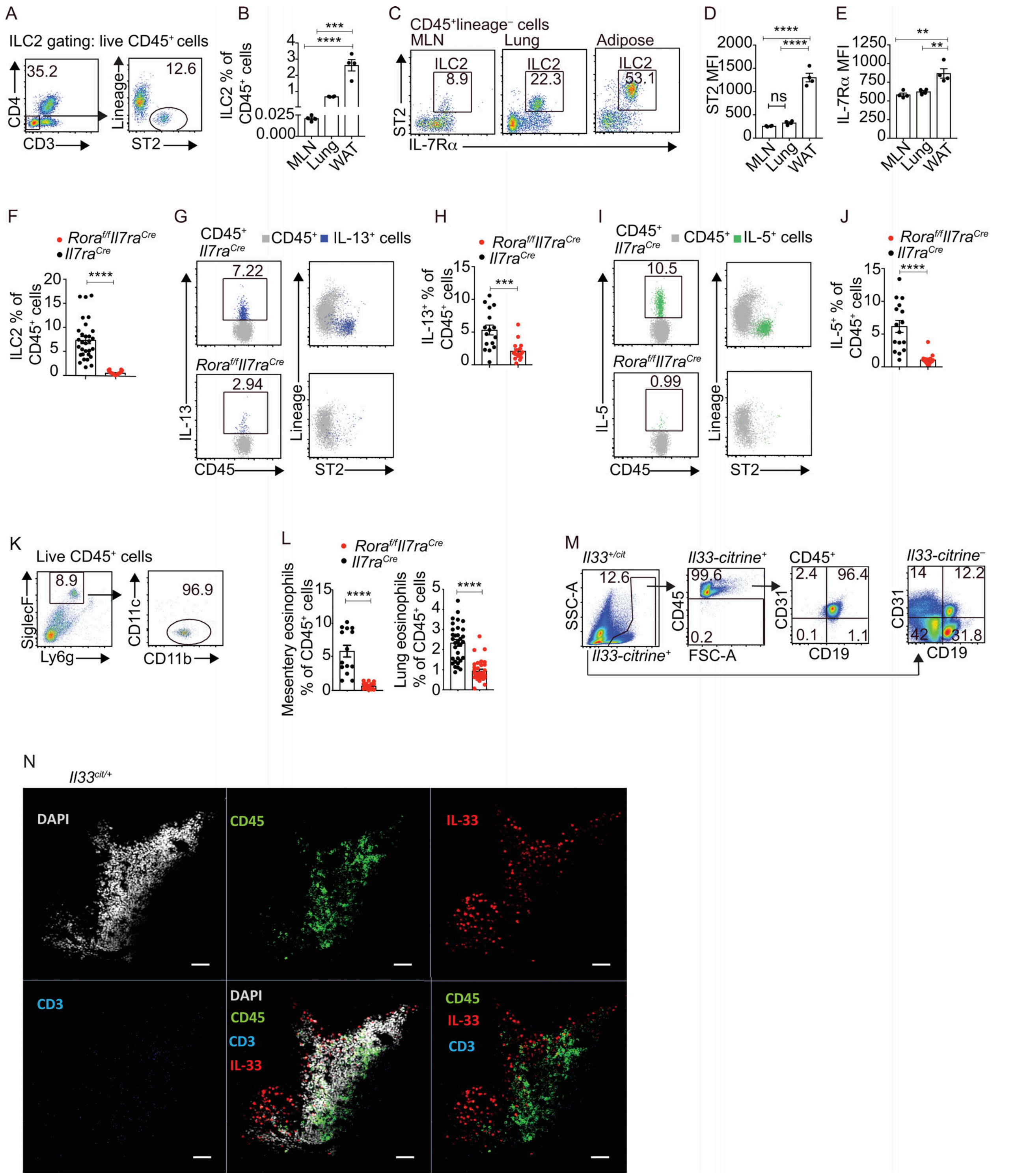

Figure S1. **Characterization of ILC2s and MSCs in WAT. (A)** Gating strategy for lineage$^-$ST2$^+$ ILC2s. **(B and C)** Flow cytometric analysis of Lin$^-$IL-7Rα$^+$ST2$^+$ ILC2 populations in the MLN, lung, and WAT (*n* = 4). **(D and E)** ST2 (D) and IL-7Rα (MFI; E) expression by Lin$^-$IL-7Rα$^+$ST2$^+$ ILC2s harvested from the MLN, lung, and WAT (*n* = 4). **(F)** ILC2 frequency in the mesentery of control (*Il7r^{Cre}*) and ILC2-deficient (*Rora$^{fl/fl}$Il7ra^{Cre}*) mice. Data are pooled from four independent experiments (*n* = 33 and 35). **(G and H)** Intracellular flow cytometric analysis showing a representative example of IL-13$^+$CD45$^+$ populations (G) and as a percentage of total CD45$^+$ cells in the mesentery of (*Il7ra^{Cre}*) and ILC2-deficient (*Rora$^{fl/fl}$Il7ra^{Cre}*) mice (H). Data are pooled from two independent experiments (*n* = 15–17). **(I and J)** Intracellular flow cytometric analysis showing a representative example of IL-5$^+$CD45$^+$ populations (I) and as a percentage of total CD45$^+$ cells in the mesentery of (*Il7ra^{Cre}*) and ILC2-deficient (*Rora$^{fl/fl}$Il7ra^{Cre}*) mice (J). Data are pooled from two independent experiments (*n* = 15–17 mice). **(K)** Gating strategy for eosinophils. **(L)** Eosinophil frequency in control (*Il7ra^{Cre}*) and ILC2-deficient (*Rora$^{fl/fl}$Il7ra^{Cre}*) mice. Data are pooled from two independent experiments (*n* = 15–17). **(M)** Phenotyping of *Il33*-citrine$^+$ cells in bone marrow. Representative data (*n* = 4 mice). **(N)** Histology of fat associated lymphoid clusters in the perigonadal fat from WT mice. Scale bars, 50 µm. Data are represented as mean ± SEM. **, $P < 0.01$; ***, $P < 0.001$; ****, $P < 0.0001$; one-way ANOVA with Tukey's post hoc test (B, D, and E), or Student's *t* test (F, H, J, and L).

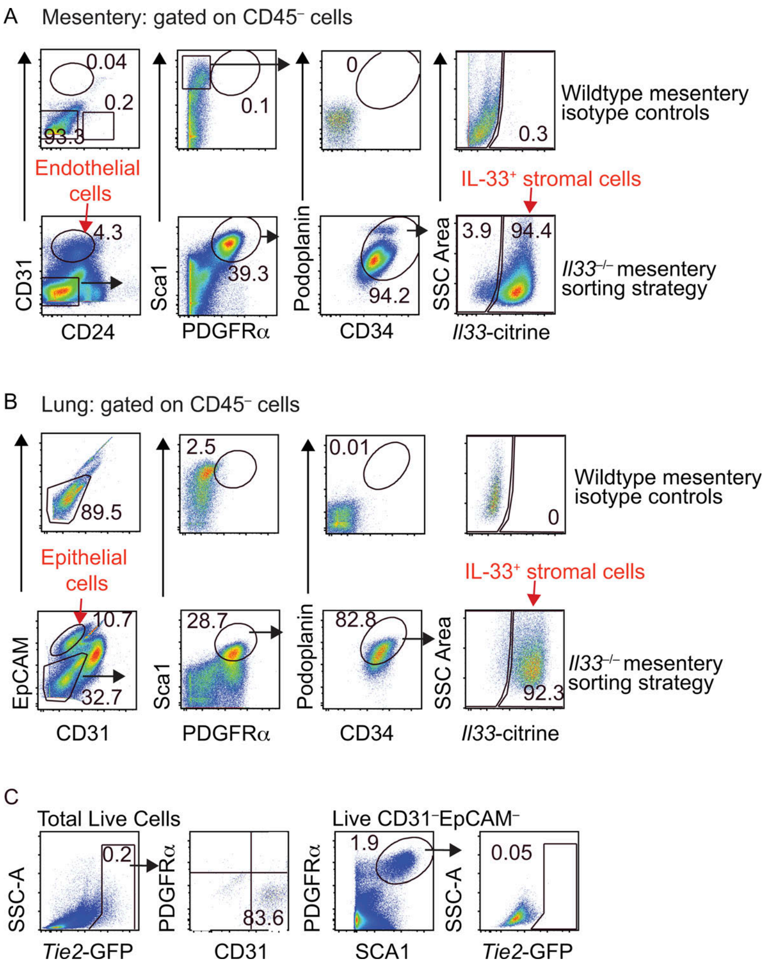

Figure S2. **Gating strategies. (A and B)** Gating strategy used to purify cell populations for RNA-seq analysis. **(C)** Flow cytometric analysis of *Tie2*-GFP+ mesentery. *Tie2*-GFP gate was determined based on WT mice (representative of two mice).

https://doi.org/10.1084/jem.20190689

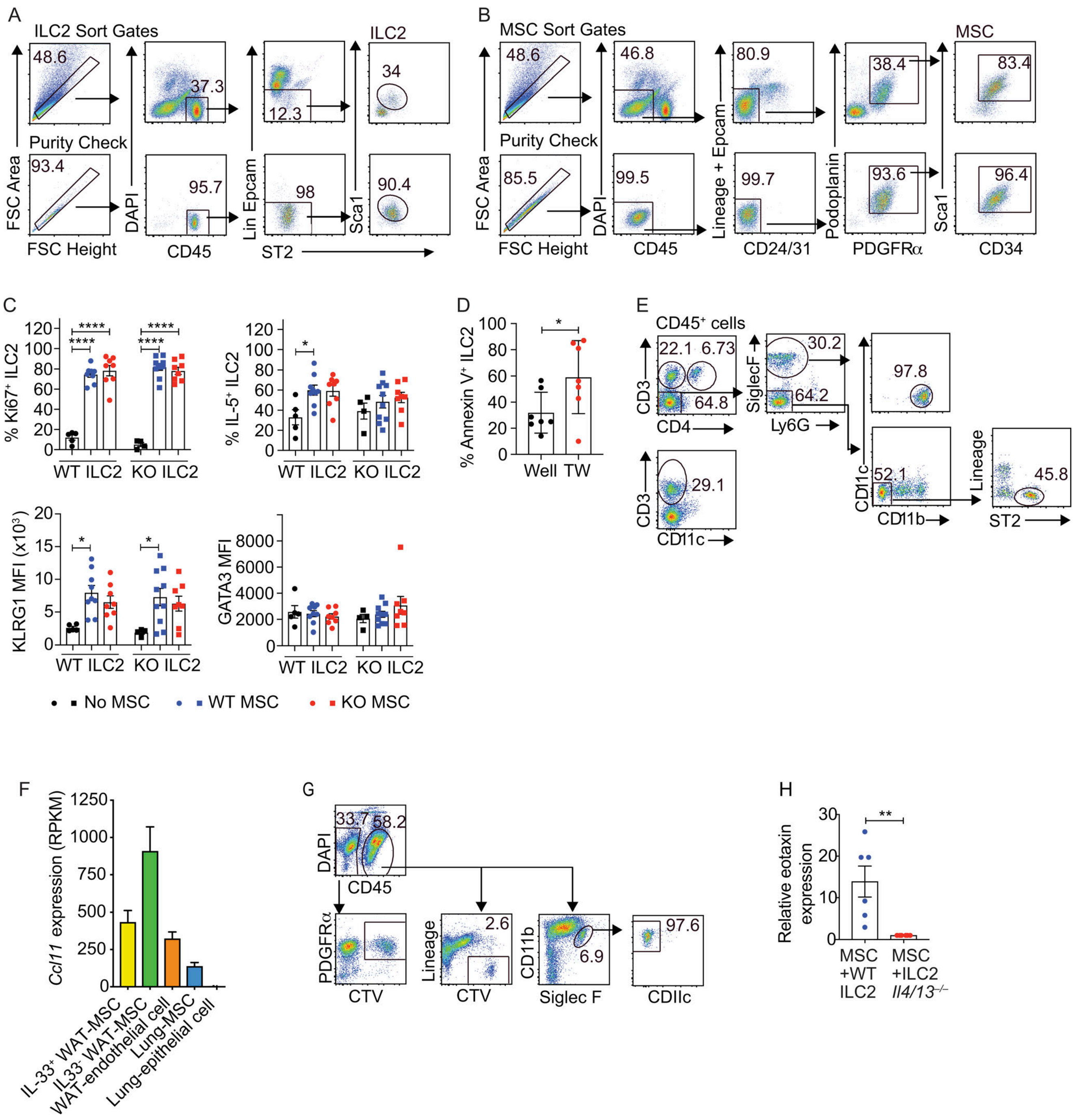

Figure S3. **Gating strategies and schematic of cross-talk between MSCs, ILC2, and eosinophils. (A and B)** Flow cytometry gating strategy and purity check for WAT-ILC2s and WAT-MSCs. **(C)** Proportion of $Ki67^+$ILC2, $IL5^+$ILC2, KLRG1 MFI, and GATA3 MFI from co-cultured WT or *Il33*$^{-/-}$ (KO) ILC2 and WT or *Il33*$^{-/-}$ WAT-MSCs, after 7 d. Cumulative data pooled from three independent experiments (n = 4–10). Cumulative data pooled from three independent experiments (n = 4–10). **(D)** Cell death of ILC2 determined on TW substrate or tissue culture plate surface by annexin V staining. Pooled data from two experiments (n = 7). **(E)** Gating strategy for T cells, eosinophils, and ILC2s. **(F)** *Ccl11* gene expression (reads per kilobase of transcript per million mapped reads; RPKM) in the tissues indicated (n = 3). **(G)** Gating strategy for CTV^+WAT-MSCs, CTV^+ILC2s and eosinophils in Matrigel plugs. **(H)** Relative eotaxin expression in supernatants from MSCs and ILC2 (WT, BALB/c WT, or BALB/c *Il4*$^{-/-}$/*Il13*$^{-/-}$) co-cultured overnight. Pooled data from six experiments. Mann–Whitney *U* test. Data are represented as mean ± SEM. *, P < 0.05; **, P < 0.01; ****, P < 0.0001.
